# Talimogene laherparepvec induced bullous pemphigoid

**DOI:** 10.1016/j.jdcr.2025.03.016

**Published:** 2025-04-02

**Authors:** Sahar Davoudi, Fade Mahmoud, Jordan Abbott

**Affiliations:** a University of Arizona College of Medicine, Tucson, Arizona; b Division of Hematology and Oncology, Banner MD Anderson Cancer Center, Gilbert, Arizona; c Division of Oncomedicine, Banner MD Anderson Cancer Center, Gilbert, Arizona

**Keywords:** autoantibody, bullous diseases, case reports, immunobiologics, melanoma, oncology

## Introduction

Advancements in immunotherapy have improved outcomes for patients with metastatic melanoma.^1^ Talimogene laherparepvec (T-VEC), an intralesional oncolytic immunotherapy, was approved for injectable and unresectable melanoma by the U.S. Food and Drug Administration in 2015.^2^ The most common adverse effects of T-VEC are pyrexia (52%), chills (48%), fatigue (32%), and nausea (30%).^2^ While cutaneous immune-related adverse events (cirAEs) in the setting of immune checkpoint inhibitors (ICIs) are widely recognized,^3^ cirAEs following T-VEC have not been well described. The only reports of cutaneous adverse events related to T-VEC are injection-site reactions and vitiligo.^2^ In this report, we present a case of bullous pemphigoid (BP) developing after T-VEC in a patient with metastatic melanoma.

## Case summary

**Fig 1:**
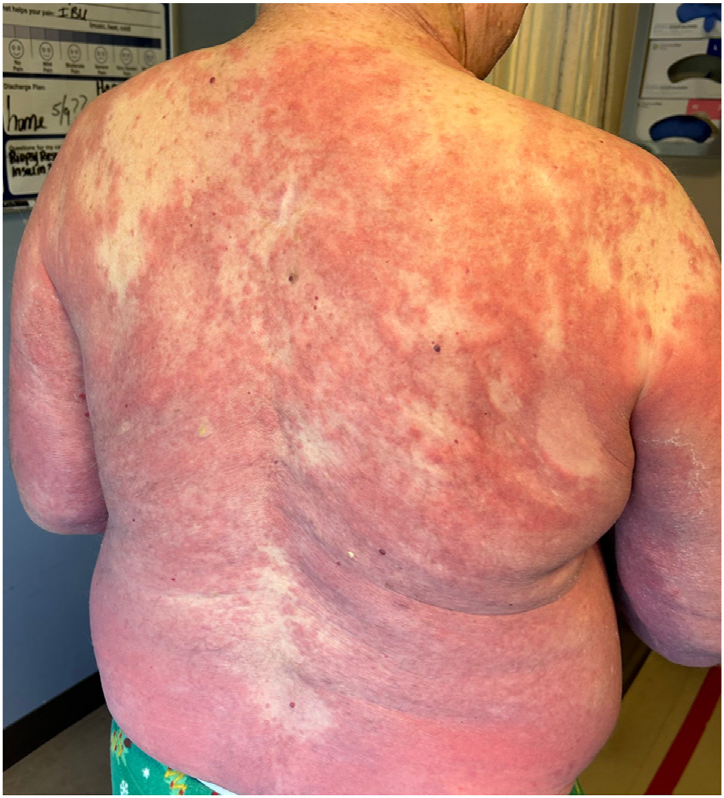
Nearly confluent erythematous patches on the back at time of initial hospital consultation.

**Fig 2:**
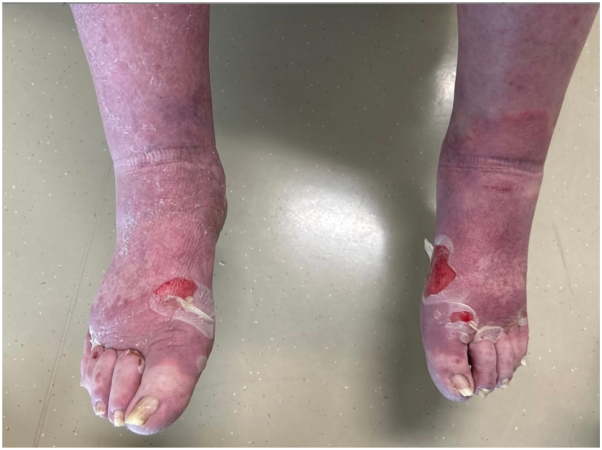
Tense and denuded bullae of the dorsal feet, at outpatient clinic follow up 2 weeks after initial eruption.

A 77-year-old male with a history of recurrent metastatic melanoma refractory to multiple ICIs developed recurrence and underwent T-VEC injection, receiving 0.5 mL concentration 10^6^ pfu/mL, at the site of recurrence. 10 days later, dermatology was consulted for inpatient evaluation. Physical exam revealed red to violaceous nearly confluent patches covering greater than 80% of the body (Fig 1). There were no bullae or areas of denuded skin and he lacked mucosal and ocular involvement. The patient stated that the rash was extremely pruritic and started 5 days prior with erythema of the right leg, which progressed quickly. At the time of evaluation, the differential diagnosis included morbilliform drug eruption, DRESS syndrome, evolving SJS/TEN, and prebullous BP. A skin biopsy revealed superficial dermal inflammation with neutrophils and eosinophils and direct immunofluorescence was positive with linear deposition of IgG and C3 along the basement membrane. Additionally, serum BP180 autoantibodies were elevated at 196 U/mL (negative, ≤14). A diagnosis of bullous pemphigoid was made. Over subsequent days, the patient developed tense bullae on the hands, wrists, legs, and feet (Fig 2). He was initially treated with prednisone 60 mg daily, doxycycline 100 mg twice a day, and clobetasol 0.05% cream twice a day. When prednisone was tapered below 20 mg, his eruption flared. Due to his metastatic cancer status, it was decided to avoid additional immunosuppression, and he was started on dupilumab 300 mg every 2 weeks. His skin cleared and he discontinued doxycycline and prednisone, while continuing dupilumab. After that, he occasionally developed small bullae on the extremities which responded quickly to twice daily clobetasol.

The patient’s initial cancer diagnosis was a stage IIIB BRAF negative 3.8 mm desmoplastic melanoma of the left cheek in 2018, for which he underwent excision and sentinel lymph node biopsy with nodal positivity. He was treated with multiple ICIs including nivolumab, pembrolizumab, and combination relatlimab/nivolumab, but developed disease recurrence on each regimen. Prior to T-VEC, he was on relatlimab/nivolumab for 7 months and had disease progression. Relatlimab/nivolumab was stopped and his last dose was 3 months prior to T-VEC initiation. While on ICI therapy, the patient developed 2 mild grade 1 cutaneous reactions. In 2022 after 3 cycles of pembrolizumab, oncology documented a pruritic skin rash successfully treated with topical triamcinolone. Additionally, in January 2024, oncology documented a rash on the forearm which also resolved with topical steroids. He was not evaluated by dermatology for these grade 1 cirAEs. Based on the description they may have represented eczematous or maculopapular eruptions, however subclinical BP cannot be excluded. His skin remained clear until May of 2024, 5 days after receiving T-VEC, when he developed the eruption for which our team was consulted. Due to the significant grade 3 cirAE following the first dose of T-VEC, oncology held additional doses. The patient underwent surgical resection of the recurrence 2 months later. Follow-up imaging showed no evidence of recurrent or metastatic disease and the patient remains on surveillance.

## Discussion

With the increase in use of novel cancer therapies like T-VEC, health care providers should be mindful of the potential for serious cutaneous adverse effects. Early detection and vigilant use of histopathologic confirmation can guide effective management, prevent worsening complications, and improve patient outcomes.

BP is a well-documented cirAE to ICI therapy,^3^ however, the role of T-VEC in the setting of ICI-induced BP has not been explored. Additionally, aside from a few reports of vitiligo and injection-site reactions,^2^ there is a paucity of literature detailing cutaneous adverse events following T-VEC. Leung et al found that combination ICI and T-VEC doubled the likelihood of cirAEs compared to ICIs alone; however, the types of cutaneous reactions were not specified.^4^

In our case, the patient developed mild grade 1 cirAEs while on ICI therapy which were appropriately controlled with topical steroids. The time frame of onset of the severe grade 3 eruption following T-VEC suggests the possibility of an interaction of T-VEC and ICI therapies. Although the mechanism behind this process has not been elucidated, we know that individuals receiving both ICIs and T-VEC are at higher risk for cirAEs than those on ICI therapy alone.^4^ In our case, we hypothesize a subclinical disease process, recognizing that IgG development takes approximately 7-14 days upon initial antigen exposure and our patient first developed cutaneous manifestations only 5 days after T-VEC initiation.^5^ In other cases of vaccine-induced BP with short latency, it has been proposed that vaccination-induced immune reactivation in predisposed individuals unmasked subclinical BP.^6^

It is important to recognize that BP may present in the prebullous phase in up to 25% of cases of immunotherapy-induced BP, as it did in our case.^7^ Cases of ICI-induced BP often have a delayed presentation; the average onset is 14-22 weeks after initiation of ICI therapy.^3^^,^^8^ In contrast to other types of drug-induced BP, these cases may persist for months after drug discontinuation.^3^ Patients with a pruritic eruption should undergo a skin biopsy with histopathological evaluation, direct immunofluorescence, and indirect immunofluorescence, if available, to ensure correct diagnosis.^9^ There are reports of ICI-related BP responding to dupilumab,^10^ and our case demonstrates success using dupilumab for BP which developed after T-VEC.

Patients undergoing T-VEC therapy should be counseled about the potential for development and exacerbation of cirAEs. Further research is necessary to better understand the cutaneous reactions to T-VEC in the setting of ICI therapy.

## Conflicts of interest

None disclosed.
